# Optimization of Chondrocyte Viability in Partially Decellularized Tracheal Grafts

**DOI:** 10.1002/ohn.404

**Published:** 2023-06-14

**Authors:** Maxwell Bergman, Jacqueline Harwood, Lumei Liu, Sayali Dharmadikhari, Kimberly M. Shontz, Tendy Chiang

**Affiliations:** 1 Department of Otolaryngology–Head & Neck Surgery, The Ohio State University Medical Center, Columbus, Ohio, USA; 2 Department of Pediatric Otolaryngology, Nationwide Children’s Hospital, Columbus, Ohio, USA; 3 Center for Regenerative Medicine, Abigail Wexner Research Institute, Nationwide Children’s Hospital, Columbus, Ohio, USA

**Keywords:** biobanking, chondrocyte viability, regenerative medicine, tissue engineering, tracheal replacement

## Abstract

**Objective.:**

Advancements in tissue-engineered tracheal replacement (TETR) show promise for the use of partially decellularized tracheal grafts (PDTG) to address critical gaps in airway management and reconstruction. In this study, aiming to leverage the immunoprivileged nature of cartilage to preserve tracheal biomechanics, we optimize PDTG for retention of native chondrocytes.

**Study Design.:**

Comparison in vivo murine study.

**Setting.:**

Research Institute affiliated with Tertiary Pediatric Hospital.

**Methods.:**

PDTG were created per a shortened decellularization protocol using sodium dodecyl sulfate, then biobanked via cryopreservation technique. Decellularization efficiency was characterized by DNA assay and histology. Viability and apoptosis of chondrocytes in preimplanted PDTG and biobanked native trachea (control) was assessed with live/dead and apoptosis assays. PDTG (N = 5) and native trachea (N = 6) were orthotopically implanted in syngeneic recipients for 1-month. At the endpoint, microcomputed tomography (micro-CT) was employed to interrogate graft patency and radiodensity in vivo. Vascularization and epithelialization were qualitatively analyzed using histology images following explant.

**Results.:**

PDTG exhibited complete decellularization of all extra-cartilaginous cells and reduced DNA content compared to control. Chondrocyte viability and nonapoptotic cell populations were improved utilizing biobanking and shorter decellularization time. All grafts remained patent. Evaluation of graft radiodensity at 1 month revealed elevation of Hounsfield units in both PDTG and native compared to host, with PDTG showing higher radiodensity than native. PDTG supported complete epithelialization and functional reendothelialization 1-month postimplantation.

**Conclusion.:**

Optimizing PDTG chondrocyte viability is a key component to successful tracheal replacement. Ongoing research seeks to evaluate the acute and chronic immunogenicity of PDTG.

Despite advancements in airway management, there remains a lack of effective surgical interventions for long-segment tracheal defects.^1,2^ At present, no suitable tracheal replacement has been identified from synthetic, autologous, or allogenic sources.^3,4^ Tissue engineering uses myriad approaches to create scaffolds to support regeneration of tissue identical to native organs. Decellularization remains the most common approach to modify allograft and xenograft tissue and has proven to be effective for clinical use with dermal matrix, small intestine submucosa, and other tissue types.^5–7^ Traditional approaches to tracheal decellularization have targeted the complete removal of all cell types to limit immunogenicity. Despite eliminating immunogenicity, these grafts have not been clinically successful, as loss of biomechanical support during decellularization results in graft stenosis and collapse.^8^ Recent advancements in tracheal decellularization have moved away from complete decellularization towards the preservation of immunoprivileged donor cartilage while removing the immunogenic extracartilaginous tissue. In animal models, partially decellularized trachea grafts (PDTG) have been found to support host-derived tracheal neotissue formation with regeneration of the tracheal epithelium and microvasculature.^3,9,10^ However, partial decellularization introduces new performance metrics for the cellular composition tracheal scaffold, in particular the integrity and viability of donor chondrocytes.

Chondrocytes are essential to cartilage and extracellular matrix (ECM) homeostasis. They alter neighboring cell functions including proliferation, self-renewal or differentiation, migration, and apoptosis.^3,4^ It can, therefore, be assumed that preservation of chondrocytes during partial decellularization could impact tracheal graft performance. Decellularization results in cell membrane injury that can ultimately compromise chondrocyte viability.

Our approach to the creation of tracheal grafts includes efforts to improve viability in allograft chondrocytes before graft implantation. This includes not only limiting decellularization reagent exposure but also takes into consideration tissue ischemia time during decellularization and graft storage conditions.^9–11^ Compared with conventional storage in −20°C, biobanking via cryopreservation increases chondrocyte viability while potentially improving cartilage integrity.^10–14^ Furthermore, we have established multimodal approaches to quantify chondrocyte viability through live/dead assay, terminal deoxynucleotidyl transferase dUTP nick end labeling (TUNEL) staining, and in vivo micro-computed tomography (microCT).^9^ In this study, our aim was to improve chondrocyte viability by modification of our decellularization protocol. We used a combination of radiographic and histologic methods to quantify the impact of our modified approach to achieve complete decellularization of the epithelium while improving chondrocyte viability in preimplanted grafts.

## Methods

### Animal Care and Ethics Statement

The animal care protocol (AR15-00090) was submitted and approved by The Institutional Animal Care and Use Committee of the Abigail Wexner Research Institute at Nationwide Children’s Hospital (Columbus, OH). All animals in this study cared for and treated according to the guidelines in the Animal Welfare Act and Public Health Service, National Institutes of Health (Bethesda, MD) in the Care and Use of Laboratory Animals (2011), US Department of Agriculture.

### Tracheal Graft Preparation

We compared 2 decellularization protocols: a conventional partially decellularization method (1×PDTG, cPDTG) and a shortened protocol that was 1/10th duration of our previous method (0.1×PDTG, sPDTG). The intent of shortening the decellularization duration was to optimize chondrocyte viability based on an established range of warm ischemia times in other animal models.^10,15–18^ sPDTG (n = 6) and syngeneic grafts (control; n = 6) were created from the proximal tracheal of female C57BL/6 J mice (n = 12), as previously published.^19,20^ Syngeneic grafts were biobanked with cryopreservation techniques.^3,21^ sPDTG (n = 6) were created by modifying prior published techniques.^3^ Briefly, we exposed grafts to decellularizing detergents, sodium dodecyl sulfate, for durations that took into consideration known murine tracheal ischemia times to limit cartilaginous penetration while also protecting the chondrocyte population. The grafts were then washed with Triton-X to remove any residual DNA. These grafts were biobanked in an identical fashion with syngeneic controls. cPDTG served as partial decellularization controls and were previously implanted into C57BL/6 J mice (n = 6). Hematoxylin and eosin (H&E) staining, live/dead assay, and TUNEL assay was then used to evaluate the chondrocyte viability and nonapoptotic chondrocyte population of native, conventional, and shortened scaffolds.^9^

### Tracheal Graft Implantation

Syngeneic grafts and sPDTG (N = 10/group) were implanted into syngeneic female C57BL/6J mice (6–8-weekold).^19,20^ First, biobanked tracheal grafts were thawed at 37°C. Next, recipient mice were anesthetized and sedated, followed by an aseptic midline incision from the sternum to the hyoid bone. The overlying strap musculature was divided, and the surrounding soft tissue was dissected from the trachea circumferentially, paying careful attention to avoid disruption of the recurrent laryngeal nerves or esophagus. The distal end of the tracheal segment was secured to the sternal notch to create a temporary tracheostomy site. The graft was implanted to the proximal native airway with 9−0 sterile nylon suture. The tracheostomy site was released with resection of a segment of native trachea followed by the distal anastomosis. Animals were carefully observed following recovery and euthanized if humane endpoint criteria were met (respiratory distress, loss of >20% preimplantation weight). At study endpoints of postoperative day 28 (POD 28), microCT was performed and animals were then euthanized using a ketamine-xylazine cocktail. Tracheal grafts were harvested and tissues were formalin-fixed, embedded, and sectioned.

### Histology

Native trachea and preimplanted sPDTG grafts were fixed in 10% neutral-buffered formalin solution, embedded in paraffin, and then longitudinally sectioned at 4 μm thickness. After decalcification, representative sections were stained with Masson’s Trichrome, Alcian Blue, and H&E (Sigma-Aldrich) to evaluate gross epithelial and submucosal tissue removal. These sections were then compared with previously stained cPDTG. The same fixation and staining procedure was then performed for syngeneic and sPTDG postimplantation.

Preimplantation chondrocyte viability and nonapoptotic chondrocyte populations were assessed with both live/dead assay and TUNEL assay, respectively. TUNEL assay was utilized to detect apoptotic chondrocytes (Sigma-Aldrich). Live/dead cytotoxicity kit (Invitrogen^™^; Thermofisher Scientific) was then performed on sPDTG and native tracheal cartilage with 0.6 μL/mL Calcein-AM and 13 μL/mL ethidium homodimer-1 in phosphate-buffered saline (PBS) (300 μL/sample). Live/dead cytotoxicity kit (Invitrogen^™^; Thermofisher Scientific) was used to stain sPDTG and native tracheal cartilage with 0.6 μL/mL Calcein-AM and 13 μL/mL ethidium homodimer-1 in PBS (300 μL/sample). Transverse sections were incubated for 15 minutes and then imaged with a confocal microscope (Zeiss LSM700). Green fluorescence indicated live cells, which approximated intracellular esterase activity, while red fluorescence represented dead cells, as ethidium homodimer-1 to indicate loss of plasma membrane integrity. Chondrocyte viability of preimplanted grafts was quantified as percentage of living cells in chondrocyte population [Live % = 100 × (N_live cells_/N_total cells_) %]^22^.

Finally, immunohistochemistry was utilized to stain chondrocytes with Sox9 antibodies. Sections were deparaffinized with xylene washes, rehydrated through graded alcohols, and equilibrated with 1× PBS solution. SOX9 primary antibody (EMD Millipore) was diluted to a working concentration of 1:400 using a blocking buffer. A primary antibody cocktail was added to sections. After overnight incubation, a secondary antibody solution (1:500 dilution of goat antirabbit immunoglobulin G with Alexa Fluor 488 [Invitrogen] in blocking buffer) was added to the section. After appropriate incubation and washing, the slides were mounted using Vectashield mounting medium with DAPI counterstain (Vector Laboratories). Images were captured using digital microscopy (Zen Blue Edition; Zeiss, Oberkochen, Germany) for analysis and archiving. Chondrocytes present in host and graft cartilage were evaluated and manually quantified using ImageJ (NIH).

### microCT

Trifoil eXplore Locus RS 80 microCT was employed to interrogate in vivo graft patency and radiodensity as an indirect measurement of calcification due to chondrocyte death.^15^ At the study endpoint on POD 28, live animals were anesthetized via inhalation (1%–3% isoflurane in room air at 1–3 L/min) and were subsequently placed in prone positioning. Airway lumens were then reconstructed and assessed 3-dimensionally using Amira (Thermo Fisher Scientific).

### Statistical Analysis

Statistical analysis was performed using GraphPad Prism 8 (GraphPad Software Inc.). The Shapiro-Wilk test was used to assess the normality of the data. Data with normal distribution were compared using Welch’s *t*-test for data with significantly different variances or using an unpaired *t*-test for data with equal variances. The Mann-Whitney nonparametric test was used for data with nonnormal distribution. Paired *t*-test was used to compare data between graft and host in each animal. Statistical difference was considered significant at *p* < .05. All experimental data were conveyed as mean ± standard deviation (SD).

## Results

### Preimplantation Histology

**Figure 1.**
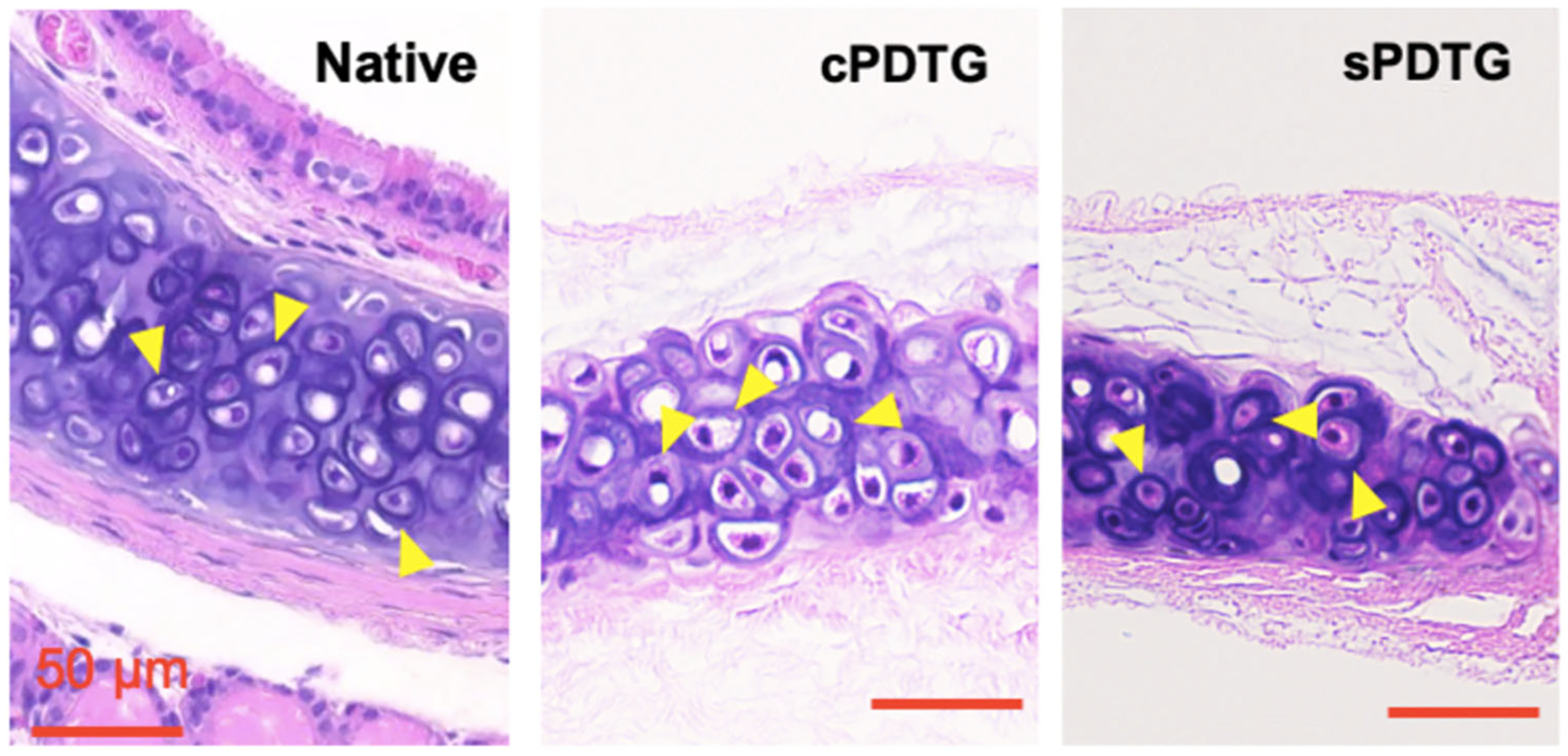
Histologic characterization of preimplanation partial decellularized trachea grafts (PDTG). Representative hematoxylin and eosin images showing both partial decellularized grafts exhibit complete removal of all extra-cartilaginous cells with preservation of underlying chondrocytes (yellow arrows).

**Figure 2.**
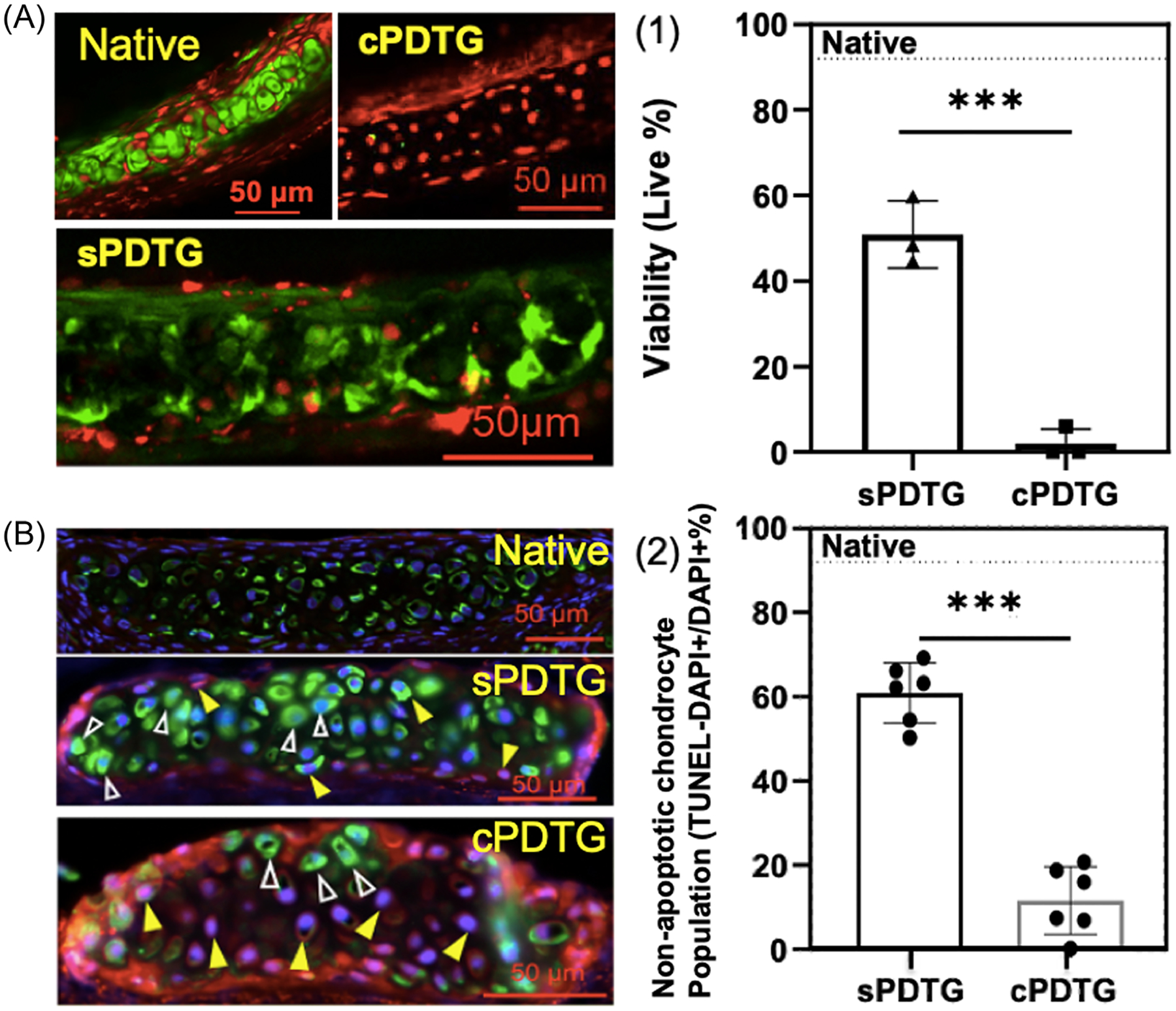
Characterization of preimplanted tracheal grafts. (A) Live/dead images of native, cPDTG, and sPDTG. (B) TUNEL assay with solid and empty triangles representing live and apoptotic cells, respectively. (1 and 2) ***p < .005. cPDTG, conventional partially decellularized tracheal grafts; DAPI, 4′,6-diamidino-2-phenylindole; sPDTG, shortened partially decellularized tracheal grafts; TUNEL, terminal deoxynucleotidyl transferase dUTP nick end labeling.

Native, cPDTG, and sPDTG were compared with H&E, TUNEL, and immunofluorescent staining. H&E staining was used to evaluate the removal of immunogenic epithelial and submucosal tissue after decellularization. Both cPDTG and sPDTG exhibited complete removal of all extra-cartilaginous cells with no evidence of residual tissue. As demonstrated in previous studies, there is conservation of underlying cartilaginous structures on Masson’s Trichrome staining.^3^ Finally, there was gross preservation of underlying ECM and chondrocytes on native, cPDTG, and sPDTG (Figure 1). Live/dead assay imaging demonstrated that chondrocyte viability of sPDTG ranged from 44.6% to 59.7%, compared with only 0%-2% seen in cPDTG from previous publications^13^ (*p* = .0031; Figure 2). Finally, TUNEL staining, an indicator of cell apoptosis, demonstrated a nonapoptotic chondrocyte population of approximately 60% compared with 10% of cPDTG (*p* < .0032; Figure 2). Overall, our shortened decellularization scaffold demonstrated 500%–600% improvement in chondrocyte survival compared with our previously published protocol.

### Postimplantation Analysis

**Figure 3.**
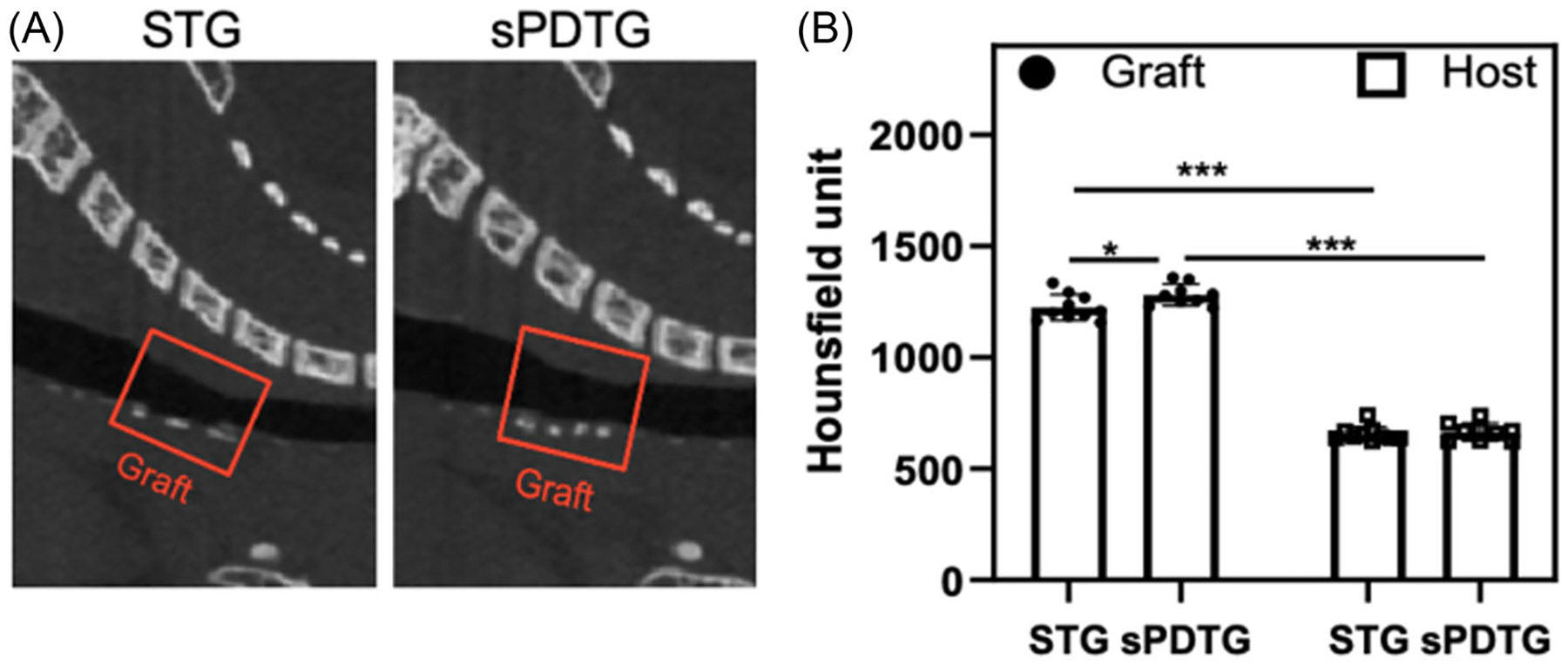
In vivo graft patency and radiodensity. (A) Sagittal micro-CT of STG and sPDTG at 28-days. Red box highlights tracheal grafts. (B) Average HU quantification. *p < .05 and **p < .0001, respectively. CT, computed tomography; sPDTG, shortened partially decellularized tracheal grafts.

At 28-day, syngeneic trachea graft STG animals survival rate was 100% (10 out of 10), and sPDTG animals survival rate was 90% (9/10). cPDTG were animals from our previous study, and we overview 11 animals’ slides and 7 microCT images for qualitative analysis. After 28 days in vivo, microCT demonstrated that all syngeneic, cPDTG, and sPDTG grafts remained patent. Hounsfield unit quantification was not available for cPDTG. Both syngeneic and sPDTG had greater calcification compared to adjacent host trachea. Specifically, sPDTG and syngeneic had Hounsfield units of 1281 and 1224 (*p* = .032), respectively, compared with adjacent host trachea of 660 (*p* < .000001; Figure 3).

**Figure 4.**
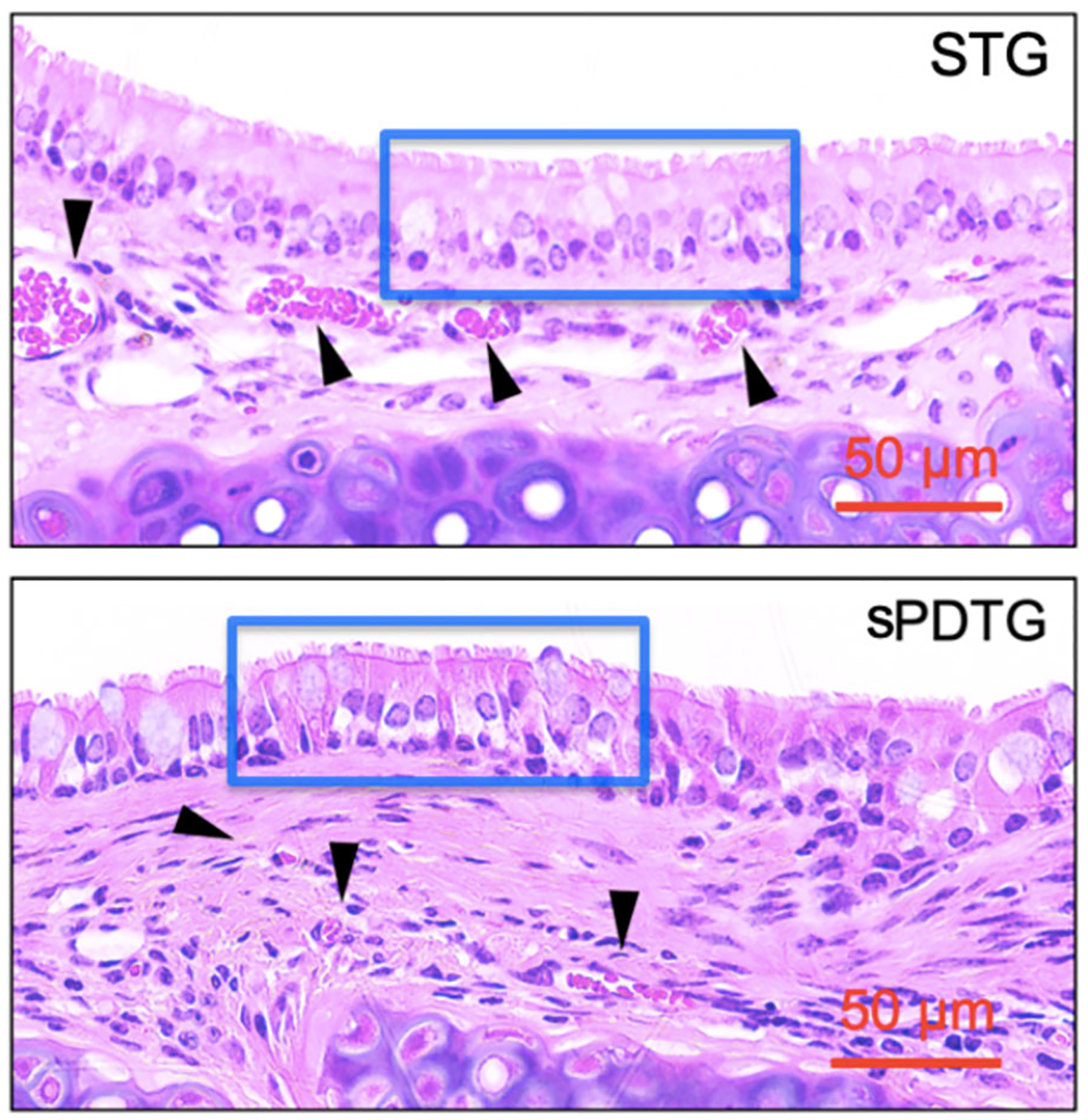
Histologic analysis of postexplant syngeneic and shortened duration decellularization graft. Representative Hematoxylin and Eosin images comparing re-epithelialization (blue outline) and neo-vascularization (black arrows) in STG and sPDTG. sPDTG demonstrates complete re-epithelialization and comparable neovascularization. sPDTG, shortened partially decellularized tracheal grafts.

Finally, postimplantation H&E showed that syngeneic and shortened partially decellularized grafts demonstrated complete regeneration of the epithelium and endothelium by the POD 28 (Figure 4).

## Discussion

Partially decellularized trachea grafts preserve underlying cartilage and support neotissue formation.^3^ We elected to modify our decellularization technique to improve chondrocyte viability based on ischemia times in other animal models.^16–18^ Given the vital importance of chondrocytes to cartilage and ECM homeostasis, we hypothesized that we could improve the decellularization process to optimize chondrocyte viability, with the hopes that these improvements would lead to improved graft healing and performance. In this study, we evaluate a shortened protocol and compare it to our previously published results.

The shortened protocol exposes explanted tissue to the same decellularization detergents as previously described.^20^ To limit chondrocyte damage and/or death, we shorted graft preparation duration equivalent to known ischemia times of explanted murine tracheas, which was approximately 1/10th our previous decellularization time. Our results were promising, as TUNEL and live/dead assays showed that chondrocytes in sPDTG increased by at least 500% preimplantation compared with our conventional protocol. Our partially decellularization protocols demonstrated complete decellularization of all extra-cartilaginous cells despite the shortened protocol, critical for preserving the nonimmunogenic properties of decellularized tissue. Moreover, on postimplant histology, sPDTG exhibited complete regeneration of tracheal neotissue. This supports the idea that we can optimize chondrocyte survival while maintaining the regenerative properties previously observed in our conventional partial decellularized grafts. In addition to functional neotissue, sPDTG were found to remain patent on MicroCT at endpoint. Notably, both sPDTG and control grafts demonstrated increase in Hounsfield unit in vivo, suggesting a surgical effect on tracheal graft calcification.

We demonstrate that alteration in decellularization protocol can improve chondrocyte viability while still removing highly immunogenic extracartilaginous cell types. These results support future efforts to focus on the impact of viable chondrocytes on tracheal neotissue formation.

This study had several limitations. First, the impact of viable chondrocytes on tracheal neotissue remains unclear; these metrics are not able to be quantified at our study endpoint. Second, microCT data from the cPDTG cohort was not available for Hounsfield unit quantification, thus limiting comparison between first- and second-generation protocols. Finally, cell function needs to be measured to fully assess chondrocyte viability of grafts in vivo.

## Conclusion

In this study, we demonstrate that shortened decellularization was associated with improved chondrocyte viability in partially decellularized trachea grafts. Optimizing PDTG chondrocyte viability is likely a key component to successful tissue-engineered tracheal replacement. Ongoing work is needed to further characterize best methods for translating partial decellularization for human use.
